# Pain perception of orthodontic treatment – A cross-sectional study

**DOI:** 10.12669/pjms.36.2.619

**Published:** 2020

**Authors:** Mazia Jawaid, Tabassum Ahsan Qadeer, Muhammad Faisal Fahim

**Affiliations:** 1 Mazia Jawaid, BDS. Registrar, Department of Orthodontics. Bahria University Medical and Dental College, Karachi, Pakistan; 2 Tabassum Ahsan Qadeer, BDS, FCPS Orthodontics. Associate Professor & Head Department of Orthodontics. Bahria University Medical and Dental College, Karachi, Pakistan; 3 Muhammad Faisal Fahim. M.Sc Statistics. Researcher, Bahria University Medical and Dental College, Karachi, Pakistan

**Keywords:** Fixed Orthodontic Appliances, Procedural Pain, Corrective Orthodontics, Dental Bonding, Orthodontic Brackets, Orthodontic Wires

## Abstract

**Objectives:**

The objectives were to evaluate the intensity of pain perceived 24 hours following insertion of fixed orthodontic appliance components i-e separators, bands and brackets.

**Methods:**

This cross-sectional study conducted at a Dental College in Karachi (July 2017-March 2018) investigated the amount of pain perceived during different stages of fixed orthodontic treatment. The inclusion criteria were healthy individuals without systemic disease, age 13-26 years, fixed orthodontic treatment candidates having healthy permanent first molars. The exclusion criteria were missing permanent first molars, patients requiring brass wire separators instead of elastomeric separators and molar tubes instead of bands, ongoing or previous periodontal disease and syndromic patients. Pain intensity was assessed in ninety eight patients using a Numeric Rating scale (NRS) at baseline (T0) before insertion, and 24 hours after insertion (T1) of a component. This procedure was repeated six times, twice each for separators, bands and brackets. Scoring was done on the NRS from 0 to 10 where 0 indicated no pain and 10 indicated worst pain possible.

**Results:**

A significant difference in perceived pain was found between baseline and after 24 hours of placement of all components (P-value 0.000). Odds ratio indicated that males were at a greater risk to feel pain than females at baseline and after 24 hours of insertion of all components. Mann-Whitney U test showed that the most painful components at baseline were bands (Mean value=0.56) and after 24 hours were brackets (Mean value 6.25).

**Conclusions:**

Significant increase in pain was noted 24 hours after insertion of separators, bands and brackets. The most painful components were bands at baseline and brackets after 24 hours of insertion. There were no significant variations in pain perception based on age. However, males perceived higher pain than females both at baseline and after 24 hours.

## INTRODUCTION

Pain due to orthodontic treatment is an important clinical problem which is generally overlooked by the dentists but is of considerable importance to the patients. Patients experience varying degree of pain and discomfort at the initial stages of fixed orthodontic treatment that negatively affects their compliance and co-operation and at times their willingness to continue the treatment.^1^-^3^ Due to this reason people have been shown to intentionally delay the orthodontic treatment, despite the esthetic and functional requirements.^1^,^4^ Orthodontic discomfort includes tension, pressure, soreness of teeth and pain^1^,^5^ and this pain occurs as a result of immediate initial compression or delayed hyperalgesia of the periodontal ligament.^3^

Factors such as past pain experiences, present emotional state, stress, age, gender and cultural differences are believed to affect an individual’s pain perception.^6^-^9^ Studies have shown that pain is generally experienced after 24 hours of insertion of different fixed treatment components including separators, and initial arch wires.^3^

Oliver and Knappman^1^ showed that 70% of the patients reported certain degree of pain while others reported that more than 90% of the patients had pain with separators, bands and brackets.^8^,^10^ Bondemark et al.^11^ concluded that after the insertion of both elastomeric and spring type separators the worst pain occurred at the second day and subsided almost completely by the fifth day. Cureton et al.^12^ concluded that elastomeric separators produced more pain as compared to springs from the second day of insertion to the fourth day.

Very few researches have compared the degree of pain perceived by the patients 24 hours after the placement of multiple fixed orthodontic treatment components. People belonging to a third world country face much more hardships as compared to the more developed countries and therefore the results of such studies if conducted in a third world country should markedly differ from the studies carried out in more developed parts of the world. Also we did not find any study conducted on Pakistani population gauging the intensity of pain perceived due to orthodontic treatment. This study will benefit the patients as they can be informed in advance regarding the level of pain that is likely to be experienced by them during treatment. In addition it will also help to improve the counseling skills at the clinician’s end. With this study we want to evaluate if there is any difference in pain perception among the different orthodontic components therefore our objectives were to evaluate the intensity of pain perceived by the patients after placement of separators bands and brackets, with identification of the most painful component, along with determination of variation in pain perception in different ages and genders.

Our null hypothesis is that there would be no difference in pain perception for the different orthodontic components used during treatment, also there would be no variation between genders and ages in pain perception.

## METHODS

This observational cross-sectional study was conducted at the Orthodontics Department of a Dental College in Karachi, Pakistan from July 2017 to March 2018. The study was approved by Ethical review board (Ref No. ERC 26/17. The inclusion criteria were healthy individuals with no systemic disease, ages between 13 and 26 years, undergoing fixed orthodontic treatment for the first time in life and having intact healthy permanent first molars. Exclusion criteria were: Patients with missing permanent first molars, those requiring brass wire separators instead of elastomeric separators and molar tubes instead of bands, history of ongoing or previous periodontal disease or bone loss (periodontal pockets>4mm), previously placed bands for any restorative procedure, syndromic patients and patients who took painkillers before or in between the different steps of the study.

The sample comprised of 98 patients (75 females and 23 males).The sample size was calculated on the basis of study conducted by Aldrees^13^ using the following equation Sample size n=[DEFF^*^Np(1-p)]/ [(d^2^/Z^2^_1-α/2_^*^(N-1)+p^*^(1-p)],keeping the population size 1000000, frequency at 90%,confidence limit 5%,confidence level at 90% and design effect as 1.Convenient consecutive sampling was used with age range of 13-26 years. The participants were grouped into 52 adolescents (13-19 years) and 46 young adults (20-26 years).^14^ The entire procedure was explained verbally to the patients and their parents and informed consent was obtained from them.

Patients were assessed with a self-administered proforma formulated using the Numeric Rating Scale (NRS). Scoring was done on the NRS from 0 to 10 where 0 indicated no pain and 10 indicated worst pain possible. The participants were instructed to encircle the numbers that they best believed represented the pain perceived by them at that time. The scoring on the NRS was first done at baseline (T0), before the fixed orthodontic appliance components were placed and the second time it was given to the participants to be filled by them 24hrs after insertion (T1) of the components. This procedure was repeated six times each for separators, bands and brackets and all the components used were from 3M Unitek. A period of one week was given in between the appointments and all the components were placed by a single operator in order to reduce operator bias.

Patients were asked to avoid the use of analgesics and were instructed to note it in the forms if used. However, none of the patients used analgesics after 24 hours of insertion of all three components. All the patients were individually called by the researcher in order to remind them to record their entries as advised and it was made sure that the participants themselves rated NRS correctly after each step so that none of the forms got discarded. The forms were returned subsequently on the next appointment.

In the first visit, operator placed the elastomeric separators inter-proximally between the permanent first molars (both mesially and distally) in all the four quadrants using separator plier. The appointment for banding of all permanent first molars was scheduled after one week of separator placement. Second molars were banded after the second form of bonding was received from the patients as these teeth were not included in the study. The bonding was done only on a single arch (Upper Arch) due to increased patient load in the OPD. Fixed appliance prescription of 0.022-slot was used and 0.012 NiTi wire was placed after bonding and secured with elastomeric o- rings in all the patients with and without crowding to maintain a similar standard. All the components were placed according to the manufacturer’s instructions and the entire procedure was performed by the same clinician in order to reduce operator bias. Out of the selected 98 patients, 58 patients had extractions in their treatment plans due to crowding. In those patients, the fixation of the brackets and grading on NRS was done two weeks after extractions so that any bias as a result of post extraction pain is eliminated.

### Statistical Analysis

SPSS version 23 was used to analyze the data with level of significance set at <0.05.Wilcoxon Sign Rank Test was performed to find any significant differences in the intensity of pain perceived at baseline and 24 hours after insertion of different fixed orthodontic appliance components.

Due to the unequal number of males and females in the study, which was due to higher number of female patients in our setting, Binary Logistic Regression Analysis was done to compare genders and age groups. Mann-Whitney U test was applied to compare the differences between independent component groups at baseline and after 24 hours following the insertion of orthodontic appliances in order to find out the most painful component at baseline and after 24 hours.

## RESULTS

After 24 hours all the components of fixed orthodontic treatment therapy were significantly painful (P-value 0.000). Table-I.

**Table-I:** Comparison of Intensity of pain perceived at baseline and 24 hours after insertion of different fixed orthodontic appliance components.

Intensity of Perceived pain	Mean	Std. Error	P-value
Separators at baseline	0.08	0.037	(0.000*)
Separators after 24 hours	5.97	0.26	
			
Bands at baseline	0.56	0.110	(0.000*)
Bands after 24 hours	4.48	0.28	
			
Brackets at baseline	0.36	0.085	(0.000*)
Brackets after 24 hours	6.26	0.27	

* Wilcoxon Sign Rank Test was applied to see the significance.

The results of Binary logistic Regression Analysis are shown in Table-II. While the age did not show any significant co-relation, odds ratio indicated that males felt more pain than females at baseline and after 24 hours of insertion of separators, bands and brackets.

**Table-II:** Age and gender variation in pain perception.

Intensity of Pain Perceived	Characteristics	Crude Odds Ratio [95% CI]	P-value
	Age (years)		
Separators at baseline	13-19	0.682 [0.260-2.41]	(0.682)
	20-26	1	
	Age (years)		
Separators after 24 hours	13-19	0.981 [0.856-1.164]	(0.981)
	20-26	1	
	Gender		
Separators at baseline	Male	1.52 [0.26-8.58]	(0.036*)
	Female	1	
	Gender		
Separators after 24 hours	Male	1.18 [0.98-1.42]	(0.007*)
	Female	1	
	Age (years)		
Bands at baseline	13-19	0.977 [0.67-1.41]	(0.902)
	20-26	1	
	Age (years)		
Bands after 24 hours	13-19	0.985 [0.85-1.13]	(0.837)
	20-26	1	
	Gender		
Bands at baseline	Male	1.88 [0.87-4.09]	(0.017*)
	Female	1	
	Gender		
Bands after 24 hours	Male	1.25 [1.03-1.52]	(0.022*)
	Female	1	
	Age (years)		
Brackets at baseline	13-19	0.825 [0.50-1.35]	(0.449)
	20-26	1	
	Age (years)		
Brackets after 24 hours	13-19	0.962 [0.82-1.12]	(0.619)
	20-26	1	
	Gender		
Brackets at baseline	Male	1.68 [0.67-4.18]	(0.026*)
	Female	1	
	Gender		
Brackets after 24 hours	Male	1.38 [1.13-1.70]	(0.002*)
	Female	1	

* Binary Logistic Regression Analysis for pain comparison between age and gender groups.

When the baseline mean pain scores of separators, bands and brackets were compared using Mann-Whitney U test; bands were found to be more painful at baseline whereas; after 24 hours of insertion, brackets were identified as the most painful component (P-value 0.000). Table-III

**Table-III:** Comparison between independent component groups at baseline and after 24 hours following the insertion of orthodontic appliances.

Follow-up	Intensity of Perceived Pain	Mean	Std. Error	P-value
Baseline	Separators	0.08	0.037	(0.000*)
	Bands	0.56	0.110	
After 24 hours	Separators	5.96	0.26	(0.000*)
	Bands	4.47	0.27	
Baseline	Separators	0.08	0.037	(0.003*)
	Brackets	0.36	0.085	
After 24 hours	Separators	5.96	0.26	(0.38)
	Brackets	6.25	0.26	
Baseline	Bands	0.56	0.11	(0.154)
	Brackets	0.36	0.08	
After 24 hours	Bands	4.47	0.27	(0.000*)
	Brackets	6.25	0.26	

* Mann-Whitney U test was applied to see the significance.

## DISCUSSION

In this study pain was evaluated using the Numeric Rating Scale (NRS) which has been used in various studies, as a subjective method of measuring pain, and has been identified as an easy and a highly reliable tool to evaluate variations in pain intensity which is comparable with VAS.^15^,^16^ Our study showed that there was a significant difference in the intensity of pain between the baseline and 24hrs of placement of all appliances. Similar results were shown by Ngan et al.^3^, Jones and Chan^5^ and many others^7^,^9^,^17^-^19^ with varying degrees of intensities in their samples. Studies have shown a peak discomfort after 24 hours of separator placement.^4^,^18^ and our sample showed similar results.

Our sample showed that patients with severe crowding showed more pain as compared to others after the separator placement. This could be due to the excessive pressure exerted by the separators when they were forced in between the tight interproximal contact points of the crowded arches. However Jones et al.^19^ found no correlation between pain and initial degree of crowding and this could be due to the use of different measurement techniques.^9^ We found out a significant difference in the pain experienced by the patients at baseline and at 24 hours after placement of arch wires. These findings are in agreement with Wilson et al.^20^ In our results bands were the least painful components after 24 hours of insertion and this is in accordance with other studies.^21^,^22^

We found significant variations in pain intensities between the two genders, as males experienced more pain when compared to females, but Ngan et al.^3^ found no significant differences in pain perception between the two genders while Jones and Chan^5^ and Feinmann et al.^23^ reported similar results as ours. Variations in pain perception have been reported in different studies. Bergius et al.^6^ and Scheurer et al.^9^ found that older subjects experienced more pain while Ngan et al.^3^ found no age variation in pain perception which is similar to our findings.

### Limitations of the study

Our study had certain limitations. Participants from the out-patient orthodontics department of only one institution were selected as the study sample and shorter follow-up period of 24 hours was observed for each component.

## CONCLUSIONS

This study concluded that a significant increase in the intensity of perceived pain was noted after 24 hours of insertion of all fixed orthodontic treatment components including separators, bands and brackets. However bands were the most painful components at baseline, while after 24hours of placement, brackets were the most painful component. No statistically significant variation in pain perception was seen when comparing the two age groups. Thus the null hypothesis was accepted. Males showed higher pain perception after the placement of all three fixed orthodontic components both at baseline and after 24 hours when compared with females. Therefore, our null hypothesis was rejected in this regard.

### Authors’ Contribution:

**MJ** conceived and designed this study, did data collection, manuscript writing, editing, compilation of results and drafting of the article, is also responsible for integrity of research.

**THQ** critical revision and final approval of the version to be published.

**MFF** did Statistical Analysis and interpretation of data

**MJ** takes the responsibility and is accountable for all aspects of the work in ensuring that questions related to the accuracy or integrity of any part of the work are appropriately investigated and resolved.
